# African particularities of sudden adult death in Togo on autopsy cases

**DOI:** 10.1016/j.heliyon.2021.e07535

**Published:** 2021-07-10

**Authors:** Tchin Darre, Toukilnan Djiwa, Mazamaesso Tchaou, Aboudoulatif Diallo, Gado Napo-Koura

**Affiliations:** a Department of Pathology, University of Lomé, Togo; b Department of Radiology, University of Lomé, Togo; c Department of Toxicology, University of Lomé, Togo

**Keywords:** Sudden death, Autopsy, Myocardial infarction, Pulmonary embolism, Togo

## Abstract

**Background:**

The purpose of the study was to determine the circumstances of occurrence of these sudden deaths, risk factors, to identify the causes of sudden death in adults at autopsy, with a view to improving prevention.

**Methods:**

This is a retrospective study of the cases of sudden death that were the subject of an autopsy in the pathology anatomy department of the University Hospital Sylvanus Olympio in Lomé from 2009 to 2018.

**Results:**

A total of 318 sudden death cases were recorded. The sex ratio (M/F) was 1.8, and the mean age was 43 ± 0.36 years. Sudden deaths were the second most common reason for autopsies after traffic accidents. The place of death was home in 76.7% of cases and in hospitals in 23.3%. Obesity was noted in 59.4%, with an umbilical adipose panicle varying between 7 and 12 cm thick. Cardiovascular causes excluding cerebral involvement (n = 173 cases, 54.40%) followed by pulmonary causes (n = 100 cases, 31.44%) were the most common cause of sudden death. The predominant cardiac pathology was infarction accounting for 32.07% of all causes of sudden death, and pulmonary embolism with 19.49% was the leading cause at the pulmonary level.

**Conclusion:**

The victims of sudden death in Togo are relatively young, predominantly male and predominantly obese. The main causes of sudden death were myocardial infarction followed by pulmonary embolism. The prevention of sudden death remains paramount, especially in the African context, where pre-hospital care is often inadequate.

## 1 Introduction

Sudden death is a natural death that occurs unexpectedly in an apparently healthy person within less than an hour of the onset of possible symptoms, after a brief agony [1]. This mode of occurrence is dramatic: with nearly 40.000 new cases/year of sudden death in France and around 300.000 cases/year in the United States, it constitutes a real public health problem whose solution will essentially be the prevention of cardiovascular diseases [2, 3]. In adults, cardiovascular, pulmonary and cerebral causes are predominant [1]. Although sudden death in adults is not exceptional in Africa, publications on this subject are rare [4, 5]. The objectives of this work are to determine the socio-demographic characteristics of sudden deaths, and to identify causes in Togolese adults over 18 years of age.

## 2 Methodology

This is a retrospective study of cases of sudden death in Togolese adults over the age of 18 who were autopsied in the pathology anatomy department at the Sylvanus Olympio University Hospital Center in Lomé (Togo). The pathology anatomy department at the Sylvanus Olympio University Hospital Center in Lomé, is the only service that performs autopsies in Togo. Togo is a small country of 56.600Km2, with a population estimated at 7.200.000 inhabitants, located between Ghana in the west and Benin in the east. All cases of autopsies performed from January 2009 to December 2018 (10 years) were included in the study. All included autopsies were performed because of the sudden onset of death in apparently healthy subjects with no known medical history; to determine the cause. The documents studied were the requisitions issued by police authorities or the gendarmerie, the autopsy register, the autopsy reports. The parameters studied were the socio-demographic characteristics of sudden-death deaths, risk factors, the causes of sudden death in adults at autopsy, with a view to improving prevention.

The autopsies were performed both at the request of the family and at the request of the judicial authorities; the costs of autopsy requests by the family are to be paid by the family.

The autopsy protocol consisted first of an external examination of the head-neck, thorax-abdomen, limbs and venous tracts. Secondly, an internal examination of the different cavities and systems was performed, including the taking of samples for histological examination. The examination of the heart consisted in taking its measurements, its weight. The external surface is assessed; then, a sagittal section is opened. The different compartments are examined. Macroscopically suspicious areas are removed, as well as systematic samples for histological examination.

All cases of myocardial infarction were of macroscopic and microscopic diagnosis. Coronary thrombosis were diagnosed histologically.

The slides were stained with hematoxylin-eosin, with Mayers' hematoxylin, and then read with a Leica type light microscope.

Myocardial infarction was defined by the presence of a circumscribed focus of ischemic necrosis.

Thrombosis was defined by the presence of a thrombus completely obstructing the vascular lumen.

Toxicological tests were performed in some cases. It should be noted that the cost of toxicology tests is the responsibility of the parents of the deceased.

The data was recorded on the Epidata 3.1 software. The data analysis was done on the software R in version 3.4.3. For the descriptive analysis, the quantitative variables were presented as mean and standard deviation and the qualitative variables as numbers and percentages.

### 2.1 Ethics approval and consent to participate

This study was approved by the “Comité de Bioéthique pour la Recherche en Santé (CBRS)” ((Bioethics Committee for Health Research) from the Togo Ministry of Health, Ref N0: 0101/2016/MS/CAB/DGS/DPLET/CBRS). This study received approval from the head of the laboratory department to be conducted.

## 3 Results

**Figure 1:**
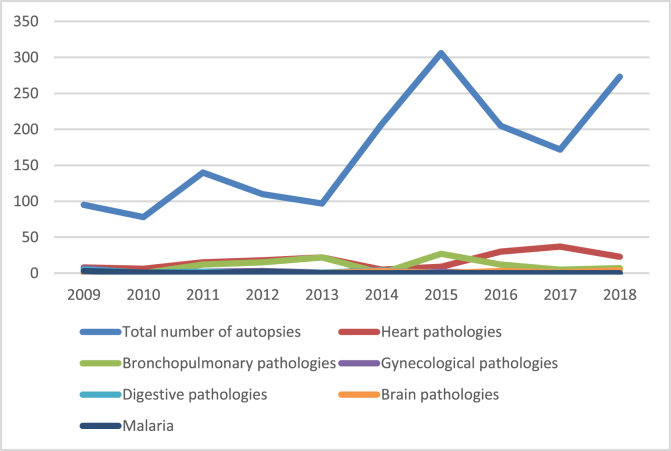
Distribution of sudden death causes by year/total number of autopsies performed.

In total, there are 318 cases of sudden death recorded an average of 31.8 cases per year. There were 202 men and 116 women, a sex ratio of 1.8. The average age was 43 ± 0.36 years, the extreme 18 years and 75 years. The average age of the Togolese population is 19.4 years with an estimated HIV prevalence of 3.2%. Sudden deaths (18.89%) were the second most common cause of autopsy after traffic accidents (74.2%) (Table 1). The place of death is at home (n = 244 cases, 76.7%) and in hospitals (n = 74 cases, 23.3%). Regarding risk factors, we noted obesity in 189 cases (59.4%), with a waist circumference between 92 and 106 cm. The causes of death were dominated by cardiovascular causes excluding cerebral involvement (n = 173 cases, 54.40%) followed by pulmonary causes (n = 100 cases, 31.44%) (Table 2, Figure 1). Acute ischemic heart disease was the leading cardiac cause, represented by acute myocardial infarction and thrombosis. Myocardial infarction (n = 102 cases, 58.96%) and accounted for 32.07% of all causes of sudden death. All cases of myocardial infarction were of macroscopic and microscopic diagnosis. These were old myocardial infarction, at least two weeks old. Early atherosclerosis of the coronary arteries was noted in 32 cases and of the aorta in 17 cases.

**Table 1:** Place of sudden death in autopsy patterns.

	Number of cases (n)	%
Public road accident	1249	74.21
Sudden adult death	318	18.89
Blows and wounds	46	2.73
Drowning	23	1.37
Works accidents	22	1.31
Suicide	19	1.13
Hanging	6	0.36
**Total**	**1683**	**100**

**Table 2:** Distribution of sudden deaths by cause.

	Number of cases (n)	%
Heart pathologies	173	54.40
Bronchopulmonary pathologies	100	31.44
Gynecological pathologies	15	4.72
Digestive pathologies	13	4.09
Brain pathologies	11	3.46
Malaria	6	1.89
**Total**	**318**	**100**

Coronary thrombosis ranked second with 33 cases (19.08%) (Table 3). All these cases of coronary thrombosis were examined histologically, with no microscopic evidence of infarction.

**Table 3:** Distribution by type of cardiac pathology.

	Number of cases (n)	%
Acute ischemic heart disease		
Infarctus	102	58.96
Thrombosis	33	19.08
Chronic ischaemic heart disease	23	13.29
Rupture of an aortic aneurysm	11	6.36
Endocarditis	03	1.73
Pericarditis	01	0.58
**Total**	**173**	**100**

There has been an increase in sudden cardiac deaths on a regular basis over the entire study period, from 7 cases in 2010, to 17 cases in 2018. This increase is mainly due to myocardial infarction, which represented 3 cases in 2009 and 12 cases in 2018.

Chronic ischaemic heart disease were found in 23 cases. Three (03) cases of endocarditis had been identified. These were abacterial thrombotic endocarditis in the mitral valve in 2 cases and in the aortic valve in one case.

We found in our series four broncho-pulmonary causes of sudden death: pulmonary embolism (n = 78 cases), bronchopneumopathies (n = 15 cases) and tuberculosis (n = 7 cases). Pulmonary embolism accounted for 19.49% of all causes of sudden death. Gynecological causes represented by erupted ectopic pregnancies in 10 cases, 4 cases of endometrial adenocarcinoma and one case of ovarian angiosarcoma. The digestive causes were haemoperitoneum (n = 11 cases) and peritonitis (n = 2cas). Haemoperitoines were secondary in 6 cases with complicated cirrhosis hepatocarcinoma, in 2 cases with adenocarcinoma of the pancreas, in 2 cases with adenocarcinoma with renal cells and one case with small cell carcinoma of the pancreas.

The cerebral causes are intracranial hemorrhages in 10 cases and brain tumors in one case. The cerebro-meningeal haemorrhages were composed of 8 cases under chronic dural hematoma, a broken aneurysm. Malaria is diagnosed in 6 cases of sudden death cases in patients who were immunocompromised for HIV.

Toxicological tests were performed in 15 cases. These toxicological tests all came back negative.

## 4 Discussion

Sudden adult death is a major problem in both developing and developed countries. In Togo in particular and in Africa in general, sudden death remains a mystery, most often leading to false accusations of poisoning or witchcraft.

We identified 318 cases in 10 years, an annual frequency of 31.8 cases. Ossei et al [5] found 1.470 cases over 9 years, an average of 163.33 cases/year. The frequencies of the African series are much lower than those of the developed countries. Indeed it is 300 000 cases/year in the United States according to Zheng et al [3]. This low frequency in our series could be explained by the still negligible requests for autopsies in cases of sudden death, related to the cultural sacredness of death in our country and the lack of systematic performance of scientific autopsies in our hospitals. It should be noted that the pathology anatomy department of the Sylvanus Olympio University Hospital is the only laboratory that performs autopsies, with the difficulty of transporting bodies from within the country. Also, the costs of autopsy examinations are to be paid by the relatives of the deceased. A national policy encouraging the performance of autopsies in cases of sudden death is necessary, by subsidizing the costs of autopsies, particularly for public health reasons. We noted a male predominance with 202 cases, a sex ratio (H/F) of 1.8. This observation is consistent with the majority of series where there was a male predominance [4, 5]. This male predominance is explained by the higher and earlier incidence of ischemic heart disease in men, a different susceptibility to ischemia, a different distribution of structural heart disease and the protective nature of estrogens [6, 7]. The average age of our patients was 43 ± 0.36 years with extremes of 9 years and 75 years. In developed countries, sudden death occurs mainly in the elderly with an average age of 68 + 20 years [2, 8]. This relatively low average age of our cases can be explained by the much lower life expectancy in developing countries in general and in Togo in particular (64 years old on average). Regarding risk factors, we noted obesity in 189 cases (59.4%). with an umbilical adipose panicle varying between 7 and 12 cm thick. The specific association between abdominal obesity and sudden death has been described. In the Paris prospective study, there was a specific risk gradient between the abdominal diameter level and sudden death, while there was no association with death from myocardial infarction [9].

Patients with a specific form of obesity, named abdominal obesity, often show clustering metabolic abnormalities which include high triglyceride levels, increased apolipoprotein B, small dense low density lipoproteins and decreased high density lipoproteins-cholesterol levels, a hyperinsulinemic-insulin resistant state, alterations in coagulation factors as well as an inflammatory profile.

Post-mortem analyses of coronary arteries have indicated that obesity (associated with a high accumulation of abdominal fat measured at autopsy) was predictive of earlier and greater extent of large vessels atherosclerosis as well as increase of coronary fatty streaks [9].

The causes of death were dominated by cardiovascular causes excluding cerebral involvement (n = 173 cases, 54.40%) followed by pulmonary causes (n = 100 cases, 31.44%).

In Togo and in Africa in general, the population is younger than in developed countries. The rural population is still large and anarchic urbanization has led to the emergence of poor socio-economic and hygienic conditions. Cardiovascular mortality increases over the years, while general mortality decreases [10]. We eat more, but we eat too much fat, too sweet, too salty, hence the appearance of vascular risk factors. Ischemic and hypertensive heart disease increases while rheumatic or nutritional heart disease is stable or decreases [11]. The causes of heart death in Africa are high blood pressure, rheumatic valve disease and infectious or postpartum cardiomyopathy [11]. Complications will be stroke, myocardial ischemia, collapse, and pulmonary embolism [10], as found in our series. Rheumatic heart disease remains present and high in most African countries with a prevalence of 25–30% [1, 10]. This drop in rheumatic heart disease is linked to the correct medical management of angina in children by appropriate and prolonged systematic antibiotic therapy; this explains the low rate of valvular heart disease in our series.

Infectious cardiomyopathies are a reflection of the sanitary conditions depreciated by overcrowding in the city favored by the rural exodus and an indicator of the insufficient level of medicalization of the health system [12]. The same is true of postpartum cardiomyopathies, which are also linked to the absence of medical specialists in remote areas of the capital. Sudden infectious death occurs after septic shock during fulminant infections [12]. Also Togo, being in malaria endemic zone, the low rate of sudden deaths by malaria access in our series may seem abnormal. This is explained firstly by the prolonged mode of evolution of malaria access which leads to death by its complications. Then the application of the WHO recommendations for the treatment of all febrile attacks by a fixed combination based on artemisinin has led to a reduction in mortality linked to malaria [13].

It should be noted that the low rate of postpartum cardiomyopathy in our context, compared with Western series, could be explained by the absence of systematic autopsy in these cases.

## 5 Conclusion

Sudden death, despite the progress of medicine, especially the means of investigation, remains a major public health issue, particularly because of its impact, but also because of its poor prognosis. In most cases, the main causes are cardiac and pulmonary. Specifying the local epidemiology will best identify the levers available to improve the management of sudden death but also better prevent the occurrence of the event. These results should lead to a health policy adapted to control cardiovascular risk factors.

## Declarations

### Author contribution statement

Tchin Darre and Gado Napo-Koura: Conceived and designed the experiments; Performed the experiments; Analyzed and interpreted the data; Contributed reagents, materials, analysis tools or data; Wrote the paper.

Toukilnan Djiwa: Conceived and designed the experiments; Contributed reagents, materials, analysis tools or data; Wrote the paper.

Mazamaesso Tchaou: Analyzed and interpreted the data; Contributed reagents, materials, analysis tools or data.

Aboudoulatif Diallo: Analyzed and interpreted the data; Wrote the paper.

### Funding statement

This research did not receive any specific grant from funding agencies in the public, commercial, or not-for-profit sectors.

### Data availability statement

Data will be made available on request.

### Declaration of interests statement

The authors declare no conflict of interest.

### Additional information

No additional information is available for this paper.
